# Packaged release and targeted delivery of cytokines by migrasomes in circulation

**DOI:** 10.1038/s41421-024-00749-x

**Published:** 2024-12-09

**Authors:** Haifeng Jiao, Xiaopeng Li, Ying Li, Ziyi Guo, Yuzhuo Yang, Yiqun Luo, Xiaoyu Hu, Li Yu

**Affiliations:** 1 State Key Laboratory of Membrane Biology, Tsinghua University-Peking University Joint Centre for Life Sciences, Beijing Frontier Research Center for Biological Structure, School of Life Sciences, Tsinghua University, Beijing, China; 2 Institute for Immunology, Tsinghua University-Peking University Joint Centre for Life Sciences, School of Medicine, Tsinghua University, Beijing, China

**Keywords:** Cell migration, Organelles, Immunology

## Abstract

In dynamic systems like the circulatory system, establishing localized cytokine gradients is challenging. Upon lipopolysaccharide (LPS) stimulation, we observed that monocytes release numerous migrasomes enriched with inflammatory cytokines, such as TNF-α and IL-6. These cytokines are transported into migrasomes via secretory carriers, leading to their immediate exocytosis or eventual release from detached migrasomes. We successfully isolated TNF-α and IL-6-enriched, monocyte-derived migrasomes from the blood of LPS-treated mice. Total secretion analysis revealed a substantial amount of TNF-α and IL-6 released in a migrasome-packaged form. Thus, detached, monocyte-derived migrasomes represent a type of extracellular vesicle highly enriched with cytokines. Physiologically, these cytokine-laden migrasomes rapidly accumulate at local sites of inflammation, effectively creating a concentrated source of cytokines. Our research uncovers novel mechanisms for cytokine release and delivery, providing new insights into immune response modulation.

## Introduction

Cytokines, as key effectors of immune cells, play a crucial role in the body’s response to infection, inflammation, and trauma^1^. Once secreted, cytokines are thought to form gradients through diffusion, leading to a diminishing concentration away from the source cells^2^. Establishing a gradient through diffusion works well in relatively static environments. However, in dynamic flow systems like the bloodstream, creating a localized gradient with a defined spatial location through diffusion alone is challenging.

Currently, multiple strategies have been identified for creating localized gradients, encompassing targeted release, attachment to the extracellular matrix, variable receptor expression, transportation by cells, and tailored degradation routes. It is believed that the localized impact of cytokines emerges from the interplay of these diverse mechanisms^3–5^.

Migrasomes are organelles that form in migrating cells, characterized by large vesicles (~2 µm in diameter) located on retraction fibers at their trailing edges^6^. These migrasomes contain numerous small intraluminal vesicles and are regulated by tetraspanin proteins^6^. For instance, overexpressing Tspan4 and Tspan9 leads to an increase in migrasome formation, while knocking out migrasome-promoting tetraspanins reduces it^7^. Migrasomes serve significant functions in various biological processes. For example, during zebrafish gastrulation, mesendodermal cells release migrasomes loaded with signaling molecules such as chemokines and growth factors, which are essential for organ morphogenesis^8^. Similarly, in chicken embryonic development, monocytes release VEGFA-enriched migrasomes along their migration paths, thereby guiding embryonic angiogenesis^9^.

Recently, we demonstrated that secretory proteins including signaling molecules such as M-CSF and CCL2 are actively transported into migrasomes, similar to the targeted neurotransmitter release in neuronal systems^10^. Secretory proteins with signaling peptides are directed to the endoplasmic reticulum (ER), transported to the Golgi complex for post-translational modifications, and sorted into secretory carriers^11,12^. In migrating cells, many of these vesicles are redirected to the cell’s rear and into migrasomes by the actin-based motor protein Myosin 5a. Once inside the migrasome, secretory carriers can fuse with the migrasomal membrane via SNARE-mediated fusion, allowing local exocytosis. If fusion does not occur rapidly, the secretory carriers and their cargo remain inside the migrasome. When the cell migrates away and the retraction fiber breaks down, these secretory proteins are released from the cell within the migrasome^10^.

In this study, we utilize a more physiological model to investigate the role of migrasomes in the targeted delivery and release of cytokines within the circulatory system. Upon lipopolysaccharide (LPS) stimulation, we found that monocytes release migrasomes enriched with inflammatory cytokines such as TNF-α and IL-6. Our results demonstrate that cell migration induces the polarization of secretory carriers toward the rear of the cell, where cytokines are encapsulated into migrasomes, facilitating their immediate exocytosis or subsequent release from detached migrasomes. Analysis of total secretion, including soluble and migrasome-packaged cytokines, reveals that migrasomes serve as a primary route for cytokine secretion in migrating cells. After LPS treatment, we observed that monocytes release cytokine-enriched migrasomes into the bloodstream, identifying migrasome-packaged cytokines as a previously unrecognized source of cytokines in circulation. Furthermore, we demonstrated that these cytokine-laden migrasomes specifically target and accumulate at local sites of inflammation, creating concentrated sources of cytokines directly at these locations. Our study suggests that the packaged release of cytokines by migrasomes may be a key mechanism for establishing local cytokine gradients in circulation.

## Results

### Monocytes release cytokine-containing extracellular particles in vivo

**Fig. 1:**
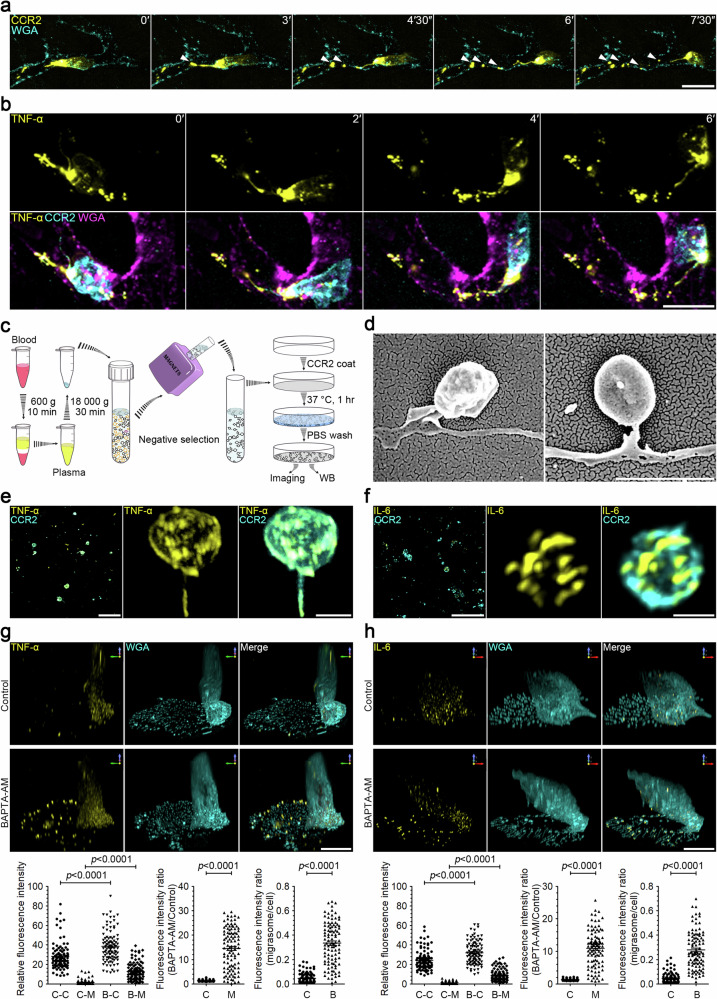
Monocytes release cytokine-containing extracellular particles in vivo, and migrasomes are the major sites of secretion in migrating cells in vitro. **a** Intravital imaging of mouse circulating monocytes and monocyte-derived extracellular particles. LPS (12 mg/kg) was injected into mice by intraperitoneal (i.p.) injection. After 2 h, CCR2-PE antibody and WGA-AF647 were injected into mice by intravenous (i.v.) injection. Intravital imaging of the mouse liver was performed to monitor blood monocytes and monocyte-derived extracellular particles. Time-lapse images were acquired at intervals of 90 s. Scale bar, 10 µm. Monocytes and monocyte-derived extracellular particles are detected with CCR2-PE antibody. WGA-F647 labels blood vessels and white arrowheads indicate free CCR2-positive particles detached from retraction fibers. **b** Intravital imaging of monocytes in mouse liver after LPS stimulation as shown in **a**. Time interval, 120 s. Scale bar, 10 μm. Monocytes and monocyte-derived extracellular particles are labeled with CCR2-PE antibody; membrane-bound TNF-α is detected with TNF-α-AF647 antibody; blood vessels are labeled with WGA-AF488. **c** Schematic illustration of the procedure for purifying monocyte-derived extracellular particles from mouse blood samples. **d** Representative scanning electron microscopy images of migrasomes isolated from blood monocytes as shown in **c**. Scale bar, 2 µm. **e**, **f** Immunofluorescence *z*-stack images of migrasomes purified from blood monocytes. Migrasomes were immunostained with antibodies against TNF-α (**e**) or IL-6 (**f**). Left, *z*-stack images were acquired by confocal microscopy. Scale bars, 10 µm. Middle and right, 3D reconstructions of enlarged migrasomes. Scale bars, 2 µm. **g**, **h** Immunofluorescence *z*-stack images of LPS-stimulated monocytes. Mouse monocytes were cultured in FN-precoated confocal dishes in the presence of 500 ng/mL LPS combined with or without 10 μM BAPTA-AM for 12 h. Cells were then immunostained with antibodies against TNF-α (**g**) or IL-6 (**h**) before visualization. *Z*-stack images were acquired by confocal microscopy, and 3D reconstructions were performed with NIS-Elements. Scale bars, 10 μm. The lower panels show statistical analysis of relative fluorescence intensities of control cell bodies (C-C), control migrasomes (C-M), BAPTA-AM-treated cell bodies (B-C), and BAPTA-AM-treated migrasomes (B-M). Data are means ± SEM. *n* > 100 cells from three independent experiments. ANOVA and post-hoc tests were used for statistical analyses. Fluorescence intensity ratio of BAPTA-AM vs control groups in cell bodies (C) or in migrasomes (M) was quantified. Fluorescence intensity ratio of migrasome vs cell body was quantified. Control (C), BAPTA-AM (B). Data are means ± SEM. Two-tailed unpaired *t*-test was used for statistical analyses.

Utilizing our recently established in vivo migrasome imaging protocol, we investigated whether monocytes can generate migrasomes in vivo. We successfully labeled monocytes with a fluorophore-conjugated anti-CCR2 antibody. In control mice, monocytes were scarcely detectable; however, following LPS stimulation, monocytes were readily observed in blood vessels, with robust formation of CCR2-positive extracellular particles (Fig. 1a; Supplementary Video S1). To determine whether cytokines, such as TNF-α, are released by circulating monocytes in vivo, we used a fluorophore-conjugated anti-TNF-α antibody to stain TNF-α in monocytes within blood vessels. Live imaging revealed distinct polarization of TNF-α towards the rear of CCR2-positive monocytes, significantly concentrating within these extracellular particles. Time-lapse imaging vividly showcased the shedding of abundant TNF-α-laden extracellular particles by these monocytes (Fig. 1b; Supplementary Video S2).

Subsequently, we isolated the monocyte-derived extracellular particles from mice blood. First, we centrifuged the blood at low speed to eliminate cells, and the crude extracellular particles isolated by high-speed centrifugation were then subjected to negative selection magnetic sorting using the EasySep Mouse Monocyte Isolation Kit (Fig. 1c). For confocal imaging analysis, we initially coated a cover glass with an anti-CCR2 antibody. This antibody-coated surface was incubated with the monocyte-derived extracellular particles, followed by washing and immunostaining (Fig. 1c). In this manner, monocyte-derived, CCR2-positive particles were captured. We observed that these CCR2-positive particles exhibited the morphological characteristics of migrasomes, including a distinctive “tail”, which is the retraction fiber (Fig. 1d). Notably, both TNF-α and IL-6 were detected in these migrasomes (Fig. 1e, f).

### Migrasomes are the major sites of secretion in monocyte cells

To better understand the mechanism underlying migrasome-packaged cytokines, we next investigated whether cultured primary monocytes could also release cytokines via migrasomes in vitro. We found that activated monocytes produce a large number of migrasomes following LPS treatment (Supplementary Fig. S1a, b). Activated monocytes secrete TNF-α and IL-6, which play important roles in innate immune responses. TNF-α is secreted in a membrane-bound form; the soluble TNF-α is cleaved from the membrane by a metalloproteinase termed TNF-alpha-converting enzyme (TACE). In contrast, IL-6 is secreted as a soluble factor. Immunostaining of activated (LPS-stimulated) monocytes with antibodies against TNF-α, TACE, and IL-6 revealed that TNF-α is indeed present in migrasomes as puncta, TACE is localized on the migrasome membrane, and IL-6 is found within the intraluminal vesicles inside migrasomes (Supplementary Fig. S1c–e). Western blotting confirmed that LPS treatment significantly enhanced the levels of TNF-α and IL-6 in both cell body and migrasomes. Moreover, TNF-α and IL-6 are not only present but also enriched in migrasomes compared to the cell body (Supplementary Fig. S1f). Next, we isolated the total membrane proteins from plasma membranes and from migrasomes. As expected, membrane-bound TNF-α is indeed markedly enriched in migrasomes (Supplementary Fig. S1g). Collectively, these findings suggest that cytokines are enriched in migrasomes.

Calcium is known to be essential for exocytosis of cytokines^13^. To accurately estimate the amount of cytokines released through the migrasome, we treated LPS-stimulated monocytes with BAPTA-AM, a cell-permeant calcium chelator, for 10 h. This treatment blocked the exocytosis of cytokines from both the migrasome membrane and the plasma membrane of the cell body. Subsequently, we stained cells with TNF-α and IL-6 antibodies, respectively. We observed that blocking exocytosis significantly increased the quantity of cytokines in the migrasome, but not in the cell body. After BAPTA-AM treatment, TNF-α and IL-6 levels increased by 14.6-fold and 10.9-fold, respectively, in the migrasome, but only by 1.41-fold and 1.35-fold, respectively, in the cell body (Fig. 1g, h), indicating that migrasomes are main exocytosis sites for cytokines.

### Secretory carriers are highly enriched in migrasome in monocytes

Next, we tested whether secretory carriers are enriched in migrasomes. Transmission electron microscopy (TEM) analysis identified numerous intraluminal vesicles inside primary monocyte-derived migrasomes (Supplementary Fig. S2a). Next, we employed antibodies targeting various markers found on secretory carriers, including Rabs, V-SNAREs, and T-SNAREs^10^. Our findings indicated the presence of several key markers for secretory carriers in migrasomes. These included Rab8a and VAMP2, which are associated with constitutive exocytosis, and Rab11a and VAMP3, which are linked to the regulated secretory pathway via recycling endosomes (Supplementary Fig. S2b–e). Additionally, we noted a significant enrichment of SNAP23, a T-SNARE, within the migrasome membrane, as shown in Supplementary Fig. S2f. Consistently, treating cells with BAPTA-AM significantly increased the number of Rab8a-, Rab11a-, VAMP2-, and VAMP3-labeled vesicles in migrasomes (Supplementary Fig. S2b–e). Together, these data suggest that secretory carriers can be enriched in migrasomes.

### Cell migration causes the polarization of secretory carriers to the rear of the cell

**Fig. 2:**
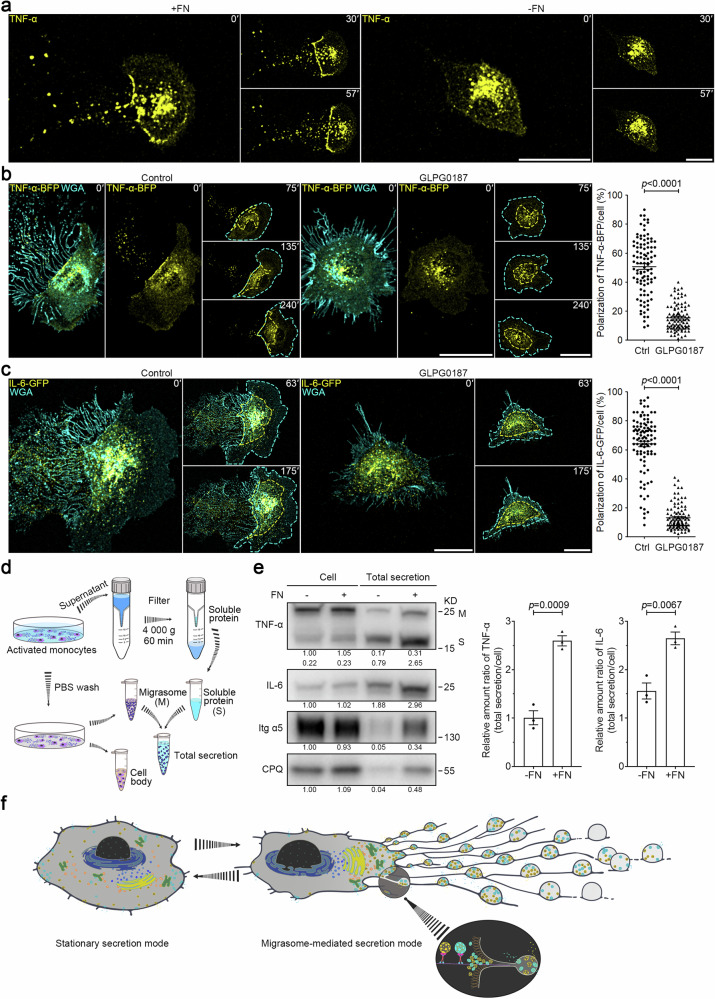
Cell migration causes the polarization of secretory carriers to the rear of the cell and drives a shift to the highly efficient, migrasome-mediated secretion mode. **a** THP-1 cells were activated in the presence of 100 ng/mL PMA for 24 h and were then plated in control or FN-precoated dishes. After TNF-α fluorescent antibody and WGA staining, time-lapse imaging was conducted. Time interval, 180 s. Scale bars, 10 μm. **b**, **c** L929 cells stably expressing TNF-α-BFP (**b**) or IL-6-GFP (**c**), treated with or without 10 μM GLPG0187, were subjected to time-lapse imaging. Time-lapse images were acquired at intervals of 15 min (**b**) or 7 min (**c**). Scale bars, 20 μm. Cyan dashed lines outline the cell body. Yellow dashed lines outline TNF-α-BFP vesicles (**b**) or IL-6-GFP vesicles (**c**), respectively. Polarization of TNF-α-BFP (**b**) or IL-6-GFP (**c**) was quantified and shown as the means ± SEM for triplicate samples of more than 50 cells. Two-tailed unpaired *t*-test was used for statistical analyses (right panel). **d** Schematic illustration of the procedure for acquiring total cellular secretion from in vitro cultured monocytes. **e** Equal numbers of mouse monocytes were seeded on control or FN-precoated dishes in the presence of 500 ng/mL LPS for 16 h. Soluble proteins in the medium were isolated using ultrafiltration, and migrasomes were purified respectively from the identical cell culture dish, as shown in **d**. Total secretion mixtures of soluble proteins and migrasomes were normalized with the numbers of cells and were then subjected to western blot analysis using the indicated antibodies. Integrin α5 (Itg α5) and CPQ are used as migrasome markers in monocytes. Both membrane-bound (M) and soluble (S) forms of TNF-α were detected by western blot analysis. Representative densitometry analysis of western blot gray values was shown. Three independent experiments were conducted. The ratio of TNF-α or IL-6 in total secretion vs cell body was quantified. Quantification is shown as the means ± SEM from three independent experiments. Two-tailed unpaired *t*-test was used for statistical analyses (right panel). **f** Model for migrating cells switching from the stationary secretion mode to the highly efficient, migrasome-mediated secretion mode.

Our in vivo data showed the release of TNF-α-laden migrasomes by monocytes into the bloodstream. To examine the release process in greater detail, we labeled phorbol-12-myristate 13-acetate (PMA)-activated THP-1 cells with a fluorophore-conjugated anti-TNF-α antibody. This antibody labeled both the plasma membrane and an intracellular vesicle population. On a control surface that does not support migration, THP-1 cells displayed a non-polarized distribution of TNF-α vesicles, as shown by time-lapse imaging. However, on a fibronectin (FN)-coated surface supportive of both migration and migrasome formation, TNF-α vesicles became noticeably polarized towards the rear of the cell and into the migrasome (Fig. 2a; Supplementary Video S3). Supporting this observation, live imaging of L929 cells expressing TNF-α-BFP or IL-6-GFP revealed that most TNF-α-BFP vesicles were positioned at the rear of the cell, with virtually no vesicles at the front. IL-6-GFP also displayed a polarized distribution, though to a lesser extent, with the majority of the vesicles at the rear and almost none at the leading edge (Fig. 2b, c; Supplementary Videos S4, S5). However, this polarization was lost when cell migration and migrasome formation were inhibited by the migration peptide inhibitor GLPG0187. Under these conditions, both TNF-α-BFP and IL-6-GFP vesicles were evenly distributed throughout the cell (Fig. 2b, c; Supplementary Videos S4, S5). It is worth noting that blocking secretion with BAPTA-AM treatment significantly increased the levels of TNF-α-GFP and IL-6-GFP in migrasomes, without affecting the levels of TNF-α-GFP and IL-6-GFP in cell bodies, further supporting that migrasomes are the main sites for exocytosis in migrating cells (Supplementary Fig. S3a, b and Videos S6, S7). Collectively, these findings suggest that cell migration can drive a shift in the secretion mode, characterized by polarized trafficking of secretory vesicles to the rear of the cell.

### Migrasome formation is required for the highly efficient, migrasome-mediated secretion mode in migrating cells

Subsequently, we aimed to determine the efficiency of the secretion in stationary cells and migrating cells. To achieve this, we developed a new protocol to quantify total cellular secretion, which includes both soluble and migrasome-packaged forms. This method required collecting the culture medium and using a filter to isolate soluble proteins via ultrafiltration. Simultaneously, we purified migrasomes from the same cell dish and introduced lysis buffer to retrieve proteins both within the migrasomes and on the filter (soluble proteins) (Fig. 2d). Using this approach, we found that monocytes cultured on FN-coated dishes, which promote migrasome formation (Supplementary Fig. S4a), displayed a 3.1-fold and 1.6-fold increase in the secretion of TNF-α and IL-6, respectively, compared to monocytes cultured in control dishes that do not support migrasome formation (Fig. 2e). Importantly, this difference in secretion was not attributed to variations in cytokine expression levels caused by the different culture conditions, as levels of TNF-α and IL-6 within cell bodies remained consistent under both conditions (Fig. 2e).

In summary, our findings suggest that when cells initiate migration, they shift to a migration-associated secretion mode. This is characterized by a polarized trafficking route, significantly boosting overall secretion levels in a manner dependent on migrasome formation (Fig. 2f).

### Tspan 9 regulates migrasome formation in monocytes

**Fig. 3:**
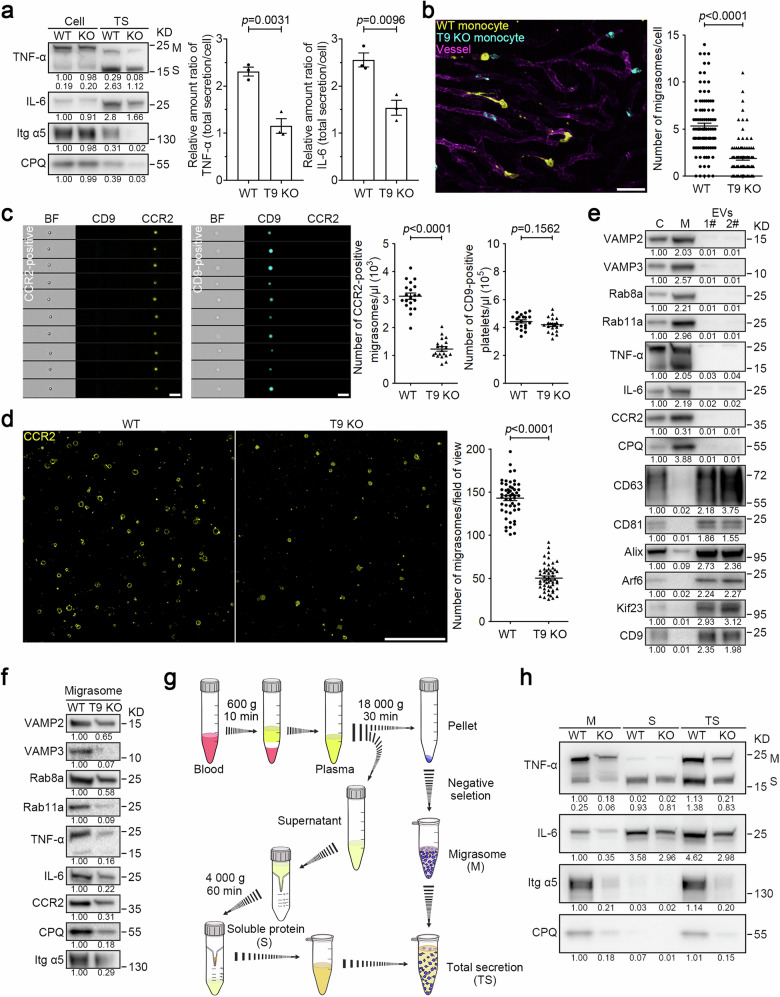
Monocytes produce fewer migrasomes in *Tspan9*^*–/–*^ mice, and T9 KO results in a marked reduction of total cytokine levels in the blood. **a** Total secretion (TS) analysis of TNF-α and IL-6 in the indicated monocytes as shown in Fig. 2d and e. Representative densitometry analysis of western blot gray values was shown. Three independent experiments were conducted. The ratio of TNF-α or IL-6 in total secretion vs cell body was quantified. Quantification is shown as the means ± SEM from three independent experiments. Two-tailed unpaired *t*-test was used for statistical analyses (right panel). **b** Equal numbers of WT and T9 KO monocytes were labeled with anti-CCR2 antibodies conjugated to different colored tags. The color-coded cells were mixed for injection into WT mice, and intravital imaging of the mouse liver was performed. Blood vessels are labeled with WGA-AF488. Time interval, 18 s. Scale bar, 20 μm. The right panel shows statistical analysis of the number of migrasomes per cell. Data are means ± SEM of more than 100 cells from three independent experiments. Two-tailed unpaired *t*-test was used to compare the datasets. **c** Diluted whole blood collected from mice with LPS stimulation was stained with CCR2-PE antibody and CD9-APC antibody for 30 min. Imaging flow cytometry analysis was performed to measure the number of monocyte migrasomes (CCR2-positive) and platelets (CD9-positive) in the blood from WT and T9 KO mice. Scale bars, 5 μm. Quantification of monocyte-derived migrasomes and platelets is shown as the means ± SEM. *n* = 20 mice from three independent experiments. Two-tailed unpaired *t*-test was used for statistical analyses (right panels). **d** Imaging analysis of CCR2-positive vesicles isolated from WT and T9 KO mouse blood samples. Scale bar, 10 μm. Quantification of monocyte-derived migrasomes is shown as the means ± SEM. *n* > 50 fields of view from 12 mice. Two-tailed unpaired *t*-test was used for statistical analyses (right panel). **e** After LPS stimulation, monocytes, monocyte-derived migrasomes, and small EVs were purified from mouse blood samples and then subjected to western blot analysis using the indicated antibodies. Lysates of monocyte cell bodies (C), monocyte-derived migrasomes (M), and small EVs (1#, 120,000× *g* and 2#, 160,000× *g*) were normalized to equal total protein loading for western blot analysis using the indicated antibodies. CD63, CD81, and Alix are used as exosome markers; Arf6 and Kif23 are used as microvesicle markers; CD9 is used as a platelet or platelet-derived EV marker. Representative densitometry analysis of western blot gray values was shown. Three independent experiments were conducted. **f** Monocyte-derived migrasomes from the indicated mice with LPS treatment were isolated from equal volumes of blood, respectively, and then analyzed by western blot analysis using the indicated antibodies. CPQ and Itg α5 are used as migrasome markers in circulating monocytes. Representative densitometry analysis of western blot gray values was shown. Three independent experiments were conducted. **g** Schematic illustration of the procedure for acquiring circulating monocyte-derived migrasomes (M), soluble proteins (S), and total secretion mixtures (TS) from mouse blood samples. **h** LPS (12 mg/kg) was injected into mice by i.p. injection. After 2 h, monocyte-derived migrasomes, and soluble proteins were isolated from equal volumes of the indicated mouse blood as shown in **g**. Migrasomes (M), soluble proteins (S), and total secretion mixtures (TS) were normalized with the volumes of mouse blood and subjected to western blot analysis using the indicated antibodies. Itg α5 and CPQ are used as migrasome markers in circulating monocytes. Both membrane-bound (M) and soluble (S) forms of TNF-α were detected by western blot analysis. Representative densitometry analysis of western blot gray values was shown. Three independent experiments were conducted.

In our previous study, we noted that members of the tetraspanin family regulate migrasome formation. Neutrophils from *Tspan9* knockout (T9 KO) mice exhibited impaired migrasome biogenesis^14^. We observed that migrasome formation is also compromised in monocytes isolated from T9 KO mice (Supplementary Fig. S4a). Furthermore, we observed that monocytes from wild-type (WT) mice exhibited total TNF-α and IL-6 secretion levels that were 2.4-fold and 1.7-fold higher, respectively, than those in monocytes from T9 KO mice (Fig. 3a). This supports our hypothesis that migrasomes play a central role in cytokine release.

### *Tspan9*^*–/–*^ mice exhibit fewer CCR2-positive particles in the blood

To investigate the role of Tspan9 in monocyte migrasome formation in vivo, we conducted three assays to compare the number of CCR2-positive, monocyte-derived particles in the blood of WT and T9 KO mice. First, we isolated monocytes from both WT and T9 KO mice, then labeled them with CCR2 antibodies conjugated to different colored tags. After mixing the color-coded cells, we injected them into WT mice. Similar to our observations in vitro, in vivo imaging revealed that monocytes from T9 KO mice produced fewer migrasomes in vivo (Fig. 3b; Supplementary Video S8). Next, to directly compare the levels of CCR2 particles in the blood of WT and T9 KO mice, we quantified the number of circulating CCR2 particles using Multispectral Imaging Flow Cytometry. Briefly, after dilution, the whole blood was stained with the CCR2 antibody to label monocytes and monocyte-derived structures, followed by imaging flow cytometry. Imaging analysis indicated that these CCR2-positive particles are small vesicles, similar to the monocyte-derived migrasomes observed in vivo. Multispectral Imaging Flow Cytometry showed that CCR2 particles were significantly reduced in the blood from T9 KO mice (Fig. 3c). Finally, we collected equal volumes of blood from WT and T9 KO mice, captured CCR2 particles on a surface coated with CCR2 antibodies, and quantified the number of captured CCR2 particles using imaging analysis. Consistently, we observed a reduction in CCR2 particles in T9 KO mice. (Fig. 3d).

### Monocyte-derived, CCR2-positive particles are migrasomes derived from monocytes

Next, we characterized the monocyte-derived particles biochemically. First, we isolated monocyte-derived particles following the protocol outlined in the schematic diagram shown in Fig. 1c. Since monocyte-derived migrasomes are CCR2-positive, the negative selection process would remove CCR2-positive particles generated by other types of cells. To verify whether monocyte-derived, CCR2-positive particles were significantly contaminated with other types of extracellular vesicles (EVs), we assessed markers classically used to analyze small EVs (including exosomes formed in multivesicular endosomes and small ectosomes formed at the plasma membrane) and large EVs. Our findings suggested that CCR2 particles were not significantly contaminated with other EV types: CCR2 particles had minimal levels of CD63, CD81 and Alix (markers for small EVs) and lacked significant amounts of Arf6 and Kif23 (microvesicle markers) (Fig. 3e). Likewise, CCR2 particles contained negligible amounts of CD9, a marker strongly linked with platelets or platelet-derived EVs in the blood (Fig. 3e; Supplementary Fig. S4b, c). In contrast, CCR2 particles, but not small EVs or microvesicles, are enriched with Carboxypeptidase Q (CPQ), a known migrasome marker^15^. These findings demonstrated that the primary distinction between migrasomes and other types of EVs is that migrasomes contain secretory carriers, a feature not reported in any other EVs. Thus, we included markers for secretory carriers, such as VAMP2, VAMP3, Rab8a, Rab11a, and secretory cytokines, such as TNF-α and IL-6, as markers for migrasomes. We found that these proteins are indeed enriched in migrasomes, but not in other types of co-isolated EVs of different sizes and densities (Fig. 3e).

In the subsequent western blot analysis on CCR2-positive particles isolated from equal volumes of blood from WT or T9 KO mice, we found that T9 KO mice showed a significant reduction in migrasome markers (Fig. 3f). It is worth noting that in T9 KO mice, the migrasomal VAMP3, Rab11a, and TNF-α were reduced more significantly than VAMP2, Rab8a, and IL-6, suggesting that the Rab11a-regulated trafficking route through the recycling endosome is more specifically directed to the migrasome than the Rab8a-regulated constitutive trafficking route. To eliminate the possibility that the reduction in these markers is due to decreased expression levels, we compared protein expression levels in monocytes isolated from both mouse types. Our findings indicated that the expression levels of these proteins were not diminished in monocytes from T9 KO mice (Supplementary Fig. S4d).

In summary, these results suggest that CCR2 particles are indeed migrasomes, and the levels of migrasome markers are significantly reduced in monocytes from T9 KO mice, which is not attributed to decreased protein expression levels.

### T9 KO results in a marked reduction of total cytokine levels in the blood

Next, we evaluated the role of migrasomes in cytokine secretion in vivo. This is particularly significant since cytokines released within migrasomes may not be detectable using standard methods like cytokine arrays. For this analysis, we isolated equal volumes of blood from WT and T9 KO mice, extracted monocyte-derived migrasomes and collected secretory proteins from the blood using ultrafiltration. We then measured the levels of TNF-α and IL-6 in these monocyte-derived migrasomes (M), in the soluble fraction (S), and in the combined sample (TS), using western blot analysis (Fig. 3g).

Our results indicated that in WT mice, ~50% of TNF-α and 24% of IL-6 were secreted in association with migrasomes (Fig. 3h). Additionally, the total secretion of TNF-α and IL-6 in WT mice was 1.8 times and 1.5 times higher, respectively, than in T9 KO mice (Fig. 3h; Supplementary Fig. S4e). These findings collectively suggest that migrasomes are a major secretion route for monocytes in vivo. Furthermore, the data reveal that a substantial amount of cytokines are released en masse within migrasomes.

### Sustained release of TNF-α from detached migrasomes

We have gathered evidence demonstrating that a significant proportion of the total inflammatory cytokines in the bloodstream are contained within circulating migrasomes. We further investigated whether these migrasome-packaged cytokines could be released after the migrasomes detach from cells.

To address this, we isolated migrasomes from TNF-α-GFP-expressing L929 cells and incubated these enriched migrasomes in a calcium-containing buffer. Time-lapse imaging showed that TNF-α-GFP-positive secretory vesicles progressively fused with the migrasome membrane, suggesting that such fusion can occur after migrasome detachment (Supplementary Fig. S5a and Video S9). To determine whether this fusion could take place under physiological conditions, we isolated monocyte-derived, CCR2-positive migrasomes from serum and then incubated them in serum for 24 h. After incubation, we immunostained the migrasomes using a TNF-α antibody. Our observations indicated that in migrasomes directly isolated from serum (without incubation), most of the TNF-α signals were intravesicular. However, after a 24-h incubation, almost all TNF-α signals appeared on the migrasome membrane, indicating the fusion of TNF-α-containing secretory vesicles with the migrasome membrane (Supplementary Fig. S5b).

For a more in-depth biochemical insight into the sustained cytokine release from migrasomes, we isolated migrasomes and soluble cytokines via filtration from equivalent serum volumes, and compared samples with and without a 24-h incubation. In the migrasome fraction (M) without incubation, TNF-α was present in both processed and unprocessed forms. After the 24-h incubation, we noted a significant reduction in TNF-α levels, regardless of its form. Similarly, the amount of IL-6 in the migrasome fraction decreased significantly post-incubation. Conversely, levels of soluble TNF-α and IL-6 (S fraction) increased after incubation, suggesting a release of these cytokines from migrasomes. When the incubation reaction was treated with BAPTA-AM, the release of TNF-α and IL-6 was inhibited, supporting the idea that cytokine release is facilitated via an SNARE-dependent fusion process (Supplementary Fig. S5c).

We found that detached migrasomes can contain TNF-α and IL-6, implying that after detachment, migrasomes might serve as vesicular carriers for signaling ligands. To determine whether migrasome-packaged cytokines are functional, we investigated whether migrasomes could convey TNF-α signaling. Leveraging the knowledge that a combination of TNF-α and the caspase 8 inhibitor zVAD can induce necroptosis in L929 cells^16^, we discovered that introducing isolated monocyte-derived migrasomes and zVAD to L929 cells effectively led to cell death (Supplementary Fig. S5d). Given that both TNF-α and TACE exist on the migrasome, we next examined whether migrasome-bound TNF-α could be cleaved by TACE to produce soluble TNF-α. We observed that TNF-α could indeed be cleaved from the migrasome, and this cleavage and migrasome-mediated cell killing could be partially inhibited by adding a TACE inhibitor. Therefore, the cytotoxic effect seems to be a combined outcome of both the cleaved free TNF-α released from the migrasome and the migrasome-associated TNF-α (Supplementary Fig. S5e). These results suggest a mechanism by which migrasomes may enable a sustained release of cytokines in the bloodstream.

### Local inflammation triggers rapid accumulation of circulating monocyte-derived migrasomes at the site of inflammation

Next, we investigated the potential physiological role of migrasome-packaged cytokines. We have previously reported that migrasomes are highly enriched with adhesion molecules, such as integrins. It is well-documented that endothelial cells undergo ‘endothelial activation’ during inflammation^17^, a process characterized by the upregulation of adhesion molecules^18^. This raises the intriguing possibility that circulating migrasomes might selectively adhere to regions of the vascular system undergoing localized inflammation.

**Fig. 4:**
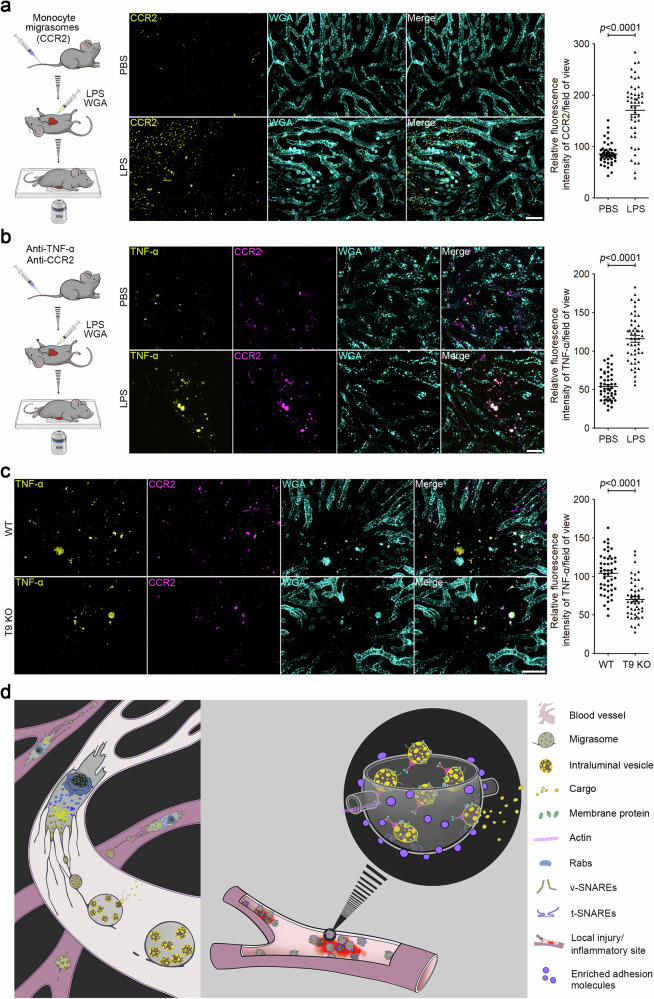
Local inflammation triggers the rapid accumulation of circulating monocyte-derived migrasomes at the site of inflammation. **a** Mice were injected with CCR2-labeled migrasomes purified from mouse monocytes by i.v. injection After 30 min, mice were anesthetized with avertin (i.p., 375 mg/kg). LPS (500 ng/mL) combined with WGA were then injected into mouse liver using a syringe. Intravital imaging of the mouse liver was immediately performed to monitor CCR2-positive migrasomes. WGA labels localized injection sites and blood vessels. Scale bar, 20 μm. The right panel shows a statistical analysis of the relative fluorescence intensity of CCR2 at injection sites. Data are means ± SEM. *n* = 8 mice from three independent experiments. Two-tailed unpaired *t*-test was used for statistical analyses. **b** LPS (12 mg/kg) was injected into mice by i.p. injection. After 2 h, fluorescent antibodies against TNF-α and CCR2 were injected into mice by i.v. injection. After localized injection of LPS (500 ng/mL) and WGA, intravital imaging of the mouse liver was immediately performed to monitor CCR2-positive migrasomes. Scale bar, 20 μm. The right panel shows a statistical analysis of the relative fluorescence intensity of TNF-α at injection sites. Data are means ± SEM. *n* = 10 mice from three independent experiments. Two-tailed unpaired *t*-test was used for statistical analyses. **c** Intravital imaging of monocyte-derived migrasomes in WT and T9 KO mice liver after LPS stimulation as shown in **b**. Scale bar, 20 μm. The right panel shows a statistical analysis of the relative fluorescence intensity of TNF-α at injection sites. Data are means ± SEM. *n* = 10 mice from three independent experiments. Two-tailed unpaired *t*-test was used for statistical analyses. **d** Model for the role of monocyte-derived migrasomes in packaged release and targeted delivery of signaling ligands to the site of inflammation.

To evaluate this hypothesis, we used a localized inflammation assay. We injected small doses of LPS into mouse livers with a syringe, enabling us to visualize the recruitment of CCR2-positive migrasomes to the injection site. For this purpose, we first purified CCR2-positive migrasomes from the blood, staining them with an anti-CCR2 antibody, followed by injecting them into the tail vein. We observed that the exogenously injected CCR2-positive migrasomes rapidly accumulated at the LPS injection site, leading to a build-up of the CCR2 signal (Fig. 4a). In contrast, a local PBS injection did not result in exogenous migrasome accumulation. This suggests that localized inflammation might facilitate the adherence and enrichment of circulating migrasomes at inflammation sites.

To determine whether endogenous monocyte-derived migrasomes, circulating in the blood, also target sites of inflammation, we first administered an i.p. injection of LPS to elevate the level of monocytes in the circulation. Two hours post LPS injection, we administered anti-CCR2 and anti-TNF-α antibodies to label the monocyte-derived migrasomes. Subsequently, we injected LPS locally and immediately proceeded with the imaging procedure. Once again, we observed that the CCR2 and TNF-α-positive migrasomes rapidly accumulated at the LPS injection sites, but not at the PBS injection sites, implying that circulating monocyte-derived migrasomes can swiftly target sites of inflammation (Fig. 4b). In contrast, the accumulation of CCR2 and TNF-α signals is significantly reduced at the LPS injection site in T9 KO mice, further confirming that migrasomes are required for the targeted delivery of cytokines at local inflammation sites (Fig. 4c).

In summary, our data point to a previously unidentified cytokine delivery mechanism. This mechanism ensures the speedy transport of various cytokines to localized inflammation sites in high concentrations via migrasomes. These migrasomes then act as a continuous local source of cytokines. Our findings shed new light on the intricate mechanisms of cytokine targeting during inflammatory responses (Fig. 4d).

## Discussion

In this study, we characterized in vivo and ex vivo that detached migrasomes are a key type of EV that serve as a major vehicle for the release of cytokines by monocytes mobilized during inflammation. We recently elucidated the molecular mechanism underlying the active transportation of signaling molecules in migrasomes. Our findings indicate that in migrating cells, secretory proteins, including signaling molecules, are actively transported into migrasomes via secretory carriers from both constitutive and regulated secretion pathways. Moreover, we provide evidence that once these carriers reach the migrasome, a portion of the cytokines is directly exocytosed, while others are released in a form packaged in the migrasome, which can later be exocytosed from detached migrasomes. Further analysis revealed that migrasomes are the primary route for secretion in migrating cells, and this migrasome-dependent secretion is significantly more efficient than the constitutive exocytosis used by stationary cells^10^.

We further showcased its occurrence in vivo, where significant amounts of cytokines are released in a migrasome-packaged form by activated monocytes. These cytokine-laden migrasomes, replete with surface adhesion molecules, circulate in the blood. When local inflammation occurs, activated endothelial cells upregulate adhesion molecules, promoting migrasome adherence. This mechanism differs significantly from the release of soluble cytokines. It ensures targeted cytokine delivery to inflammation sites, achieving higher local concentrations than soluble cytokines in the bloodstream. Additionally, since multiple cytokines can be packed into a single migrasome and released simultaneously, migrasomes could convey a synergistic combination of signals, such as TNF-α and IL-6. Moreover, cytokine release from detached migrasomes may be gradual, positioning migrasomes as sustained-release entities. We speculate that this migrasome-mediated cytokine release plays pivotal role in immune responses.

In this study, we focus on monocytes, while recognizing that migrasomes can be generated by various cell types, including neutrophils, fibroblasts, and circulating tumor cells^10,14,19^. Since the secretion of signaling molecules is essential for the functions of many of these cells, we speculate that this mechanism may be conserved across different cell types. This warrants further investigation. Additionally, this study concentrates on circulation; however, monocytes can also generate migrasomes outside of blood vessels. For instance, our previous study demonstrated that monocytes can deposit VEGFA-enriched migrasomes in the chorioallantoic membrane to guide and facilitate angiogenesis^9^. It is well established that upon activation in response to inflammation or injury, monocytes migrate from the bloodstream into tissues and differentiate into macrophages or dendritic cells. Therefore, we hypothesize that monocytes may also deposit migrasomes during this process to support their functions. The role of migrasomes in these physiological contexts remains to be investigated.

## Materials and methods

### Reagents and antibodies

FN (#PHE0023), WGA (#W7024), Puromycin (#A1113803), Prolong Live Antifade Reagent (#P36975), and Lipofectamine 3000 (#L3000001) were purchased from Thermo Fisher Scientific. BAPTA-AM (#A1076), Propidium Iodide (#81845), and LPS (#L2630) were purchased from Sigma–Aldrich. GLPG0187 (#HY-100506) and JG26 (#HY-120852) were purchased from MCE. Z-VAD-FMK (#S7023) was purchased from Selleck. Vigofect (#T001) was purchased from Vigorous. G418 (#E859) was purchased from Amresco. Hygromycin B (#10843555001) was purchased from Roche.

Anti-Rab8a (#ab188574), anti-Cellubrevin (#ab5789), anti-SNAP23 (#ab4114), anti-CD9 (#ab223052), anti-CD63 (#ab217345), anti-ARF6 (#ab131261) and anti-TACE (#ab2051) antibodies were from Abcam. Anti-Rab8a (#6975), anti-Rab11 (#5589), anti-VAMP2 (#13508), anti-TNF-α (#11948), anti-IL-6 (#12912), anti-Itg α5 (#4705) and anti-Alix (#92880) antibodies were from Cell Signaling Technology. Anti-Cellubrevin (#ET7108-31), anti-CCR2 (#ET1611-65) and anti-CD81 (#ET1611-87) antibodies were from HuaAn Biotechnology. Anti-GAPDH (#60004-1-Ig), anti-VAMP7 (#22268-1-AP), and anti-KIF23 (#28587-1-AP) antibodies were from the Proteintech Group. Anti-CCR2 (#MA5-42780) and anti-CD9 (#MA5-31980) antibodies were from Thermo Fisher Scientific. Anti-CPQ (#HPA023235-100UL) antibody was from Sigma–Aldrich.

PE anti-mouse CCR2 (#150609), APC anti-mouse CCR2 (#150627), PE anti-mouse CD115 (#135505), APC anti-mouse Ly6C (#128015), FITC anti-mouse CD9 (#124807), APC anti-mouse CD9 (#124811), Alexa Fluor 488 anti-mouse TNF-α (#506313), PE anti-mouse TNF-α (#506104) and Alexa Fluor 647 anti-mouse TNF-α (#506314) antibodies were from BioLegend. Goat anti-rabbit IgG (#111-035-003) and goat anti-mouse IgG (#115-035-003) were from Jackson.

### Cells

Mouse bone marrow monocytes (BMMs) were acquired from WT or T9 KO C57BL/6 mice as previously described^20^ using the EasySep mouse monocyte isolation kit (#19861, STEMCELL Technologies) according to the manufacture’s protocols. Briefly, bone marrow cell suspensions (1 × 10^8^ cells/mL) were incubated sequentially with isolation cocktail components and Dextran RapidSpheres. Unwanted cells (T cells, B cells, NK cells, DCs, etc.) were labeled with antibodies coupled to magnetic particles, and separated from unlabeled cells (monocytes) by a magnet. For purity assessment, cells stained with CD115 and Ly6C antibodies were analyzed by flow cytometry. BMMs were grown in RPMI 1640 medium (#C11875500BT, Gibco) supplemented with 10% FBS, 2 mM GlutaMAX, and 100 U/mL penicillin-streptomycin in 5% CO_2_ at 37 °C. BMMs were activated with 500 ng/mL LPS for 12–24 h.

THP-1 cells were grown in DMEM (#C11995500BT, Gibco) supplemented with 10% FBS (#04-001-1 A, Biological Industries), 2 mM GlutaMAX (#35050-061, Gibco) and 100 U/mL penicillin-streptomycin (#GNM15140, GENOM). Cells were activated in the presence of 100 ng/mL PMA for 24 h.

L929 cells were cultured in DMEM (#C11995500BT, Gibco) supplemented with 10% FBS (#04-001-1 A, Biological Industries), 2 mM GlutaMAX (#35050-061, Gibco) and 100 U/mL penicillin-streptomycin (#GNM15140, GENOM). Cells were cultured at 37 °C in an incubator with 5% CO_2_.

### Mice

CRISPR/Cas9 was used to generate *Tspan9*^*–/–*^ C57BL/6 mice as previously described^14^. The sgRNA sequence designed to target exon 4 of mouse *Tspan9* was 5′-GAAGGTGGCGAAGTTGCCTT-3′. The following primers were used for genotyping by PCR: *Tspan9*^*–/–*^ forward (GCTGCCTCGTCCCATTTACT) and *Tspan9*^*–/–*^ reverse (ACGCTGAGAAGCAGACACTT). Gender- and age-matched WT C57BL/6 mice were used as controls for T9 KO animals. WT C57BL/6 mice were from the Animal Facility at Tsinghua University. Animals were maintained in a light- and temperature-regulated room under specific pathogen-free conditions. Animal studies and experiments were conducted under protocols approved by the Institutional Animal Care and Use Committee at Tsinghua University and were performed following the guidelines of the Laboratory Animal Research Center of Tsinghua University.

### Cell transfection

Vigofect was used for cell transfection according to the manufacturer’s manual. Stably expressing cell lines were generated using the PiggyBac Transposon Vector System as previously described^14^. Briefly, various proteins were cloned into pB-CAG (transposon vector) as the expressing plasmid backbone. The pB-CAG constructs combined with pBASE (transposase vector) were co-transfected into cells at a ratio of 1:3 using the above Vigofect transfection protocol. After 24 h, the cells were treated with 600 µg/mL G418 or 200 µg/mL hygromycin B for selection (3–5 days). Single cells were sorted into 96-well plates by flow cytometry. These single-cell clones were cultured and expanded, followed by confocal analysis.

### Cell imaging and image analysis

10 µg/mL FN was used to precoat confocal dishes at 37 °C for at least 1 h. For confocal snapshot images, cells were cultured in FN-precoated confocal dishes for 10–12 h, and imaged by a NIKON A1RSiHD25 laser scanning confocal microscope at 1024 × 1024 pixels. *Z*-stack imaging of cells and migrasomes was performed with a NIKON A1 microscope.

For long-term time-lapse imaging, cells were grown in FN-precoated confocal dishes for 4–6 h before imaging. Cells were then maintained in the living cell system (37 °C, 5% CO_2_), and monitored by a NIKON A1 microscope. NIS-Elements Analysis 5.4 software was used to deconvolute images acquired by the NIKON A1 microscope. *Z*-projection and 3D reconstruction were performed with NIS-Elements 5.4. Images were processed using ImageJ and Imaris software 8.1.4, and statistical analyses were conducted by GraphPad Prism 8.

### Intravital imaging

Spinning disk microscopy (Perkin Elmer) was used to acquire time-lapse multiple-view *z*-stack intravital images as previously described^14^. Briefly, to image circulating monocytes, LPS (12 mg/kg) was injected into mice by i.p. injection. C57BL6/J mice were injected with 5 μg WGA and 1–2 μg fluorophore-conjugated antibody by intravenous injection at 2–4 h post LPS stimulation. WGA and CCR2 antibodies were used to label vessels and monocytes, respectively. After 5 min, avertin (375 mg/kg) was i.p. injected into mice to induce anesthesia. Subsequently, the anesthetized mice were anatomized to expose the liver, and the blood vessels on the surface were monitored by spinning disk microscopy.

For the combined imaging of WT and T9 KO monocytes, monocytes were isolated from WT and T9 KO mice and incubated with PE anti-mouse CCR2 antibody and APC anti-mouse CCR2 antibody, respectively. After washing with PBS, the WT and T9 KO monocytes labeled with CCR2 antibodies conjugated to different colored fluorescent proteins were combined in equal amounts and injected into the spleen of C57BL6/J mice which had been intravenously injected with 5 mg WGA. After being anesthetized, the mice were anatomized to expose the liver, and the blood vessels on the surface were monitored by spinning disk microscopy. Images were processed using ImageJ and Imaris software 8.1.4, and statistical analyses were conducted by GraphPad Prism 8.

### Scanning electron microscopy

Purified migrasomes were placed on a wafer which was coated with anti-CCR2 antibody for 2 h. After washing with PB buffer, samples were fixed with 2.5% glutaraldehyde for 1 h. Samples were then washed in PB for 10 min, and incubated with 1% osmium tetroxide/1.5% potassium ferricyanide for 30 min. After washing in distilled water, samples were dehydrated in an ethanol series (50%, 70%, 80%, 90%, 100%, 100%, 100%; 2 min each). The samples were dried in a critical point dryer (Leica EM CPD300). A 10-nm gold layer was sputtered onto the surface of the samples, which were then observed under an FEI Helios NanoLab G3 UC scanning confocal microscope.

### TEM

Cells were grown in 35-mm dishes precoated with FN (10 µg/mL). After 10–12 h, cells were pre-fixed with a mixture of growth medium and 2.5% glutaraldehyde (1:1 ratio) for 5 min at room temperature. Cells were further fixed with 2.5% glutaraldehyde in PB buffer for 2 h at room temperature, washed three times with PBS, and dehydrated through a graded ethanol series (50%, 70%, 90%, 95%, and 100%) for 8 min each. Samples were infiltrated with and embedded in SPON12 resin. Following polymerization was conducted at 60 °C for 48 h. 70 nm-thick ultrathin sections were cut using a diamond knife and collected on Formvar-coated copper grids (100 mesh). The sections were double stained with uranyl acetate and lead citrate. After being air-dried, samples were examined using a transmission electron microscope H-7650B at an acceleration voltage of 80 kV.

### Isolation of migrasomes from cultured cells

Crude migrasomes were collected by differential centrifugation as previously described^15^. Briefly, cells and migrasomes from 15-cm dishes were carefully harvested into 50 mL tubes after trypsin digestion. All subsequent steps were performed at 4 °C. After initial double centrifugation at 600× *g* for 10 min at 4 °C, the supernatant was subjected to a further spin at 2000× *g* for 20 min at 4 °C to eliminate cell bodies and large debris. The crude migrasomes were pelleted by centrifugation at 18,000× *g* for 30 min at 4 °C.

High-purity migrasome isolation was performed by iodixanol-sucrose (Sigma–Aldrich, LYSISO1) density gradient centrifugation following the protocol that we set up previously^21^. Briefly, the crude migrasome pellet was resuspended in 800 µL of a buffer mixture (400 µL extraction buffer combined with 400 µL 10% Optiprep) and then layered onto a multistep Optiprep dilution gradient for centrifugation at 150,000× *g* for 4 h at 4 °C. The gradient consisted of steps at 50% (500 µL), 40% (500 µL), 35% (500 µL), 30% (500 µL), 25% (500 µL), 20% (500 µL), 15% (500 µL), 10% (500 µL), and 5% (500 µL), topped with crude migrasomes (5%, 800 µL). After centrifugation, samples were collected from the top, fraction by fraction (500 µL each). Fractions 4, 5, and 6 were each mixed with 500 μL PBS, respectively, and then centrifuged at 18,000× *g* for 30 min at 4 °C. The resulting pellets were washed with PBS and re-centrifuged at 18,000× *g* for 30 min to pellet the migrasomes. The samples were then ready for downstream applications, including western blot and TEM analyses.

### Isolation of migrasomes from mouse blood

LPS (12 mg/kg) was injected into mice by i.p. injection. After 2 h, blood was collected from mice by cardiac puncture and mixed with the same volume of blood collection buffer (10 mM EDTA in PBS) on ice. The blood mixture was centrifuged at 600× *g*, 4 °C for 10 min followed by 2000× *g*, 4 °C for 20 min to remove the blood cells. Crude migrasomes were then collected as the pellet by centrifugation at 18,000× *g* for 30 min at 4 °C.

To purify monocyte-derived migrasomes, crude migrasomes were negatively selected by magnetic sorting using the EasySep mouse monocyte isolation kit. Briefly, the pellet containing the crude migrasome fraction was resuspended and then incubated sequentially with isolation cocktail components and Dextran RapidSpheres. Unwanted components were labeled with antibodies coupled to magnetic particles, and separated from monocyte-derived migrasomes by a magnet.

For imaging analysis, anti-CCR2 antibody was coated onto a cover glass or a wafer. The crude migrasome preparation was incubated with the antibody-coated surface, followed by washing and immunostaining. For western blot analysis, the crude migrasome preparation was incubated in antibody (CCR2)-precoated 6-cm dishes, followed by washing and lysing by 2.5% SDS lysis buffer.

### Isolation of small EVs from mouse blood

The isolation of microvesicles and exosomes was performed as previously described with minor modifications^22^. The blood mixture was firstly centrifuged at 600× *g*, 4 °C for 10 min followed by 2000× *g*, 4 °C for 20 min to remove the blood cells. Crude migrasomes were then removed as the pellet by centrifugation at 18,000× *g* for 30 min at 4 °C. The supernatant was then centrifuged at 120,000× *g* (1#) or 160,000× *g* (2#), 4 °C for 2 h to collect the pellet, which is enriched with microvesicles or exosomes.

### Multispectral imaging flow cytometry

LPS (12 mg/kg) was injected into mice by i.p. injection. After 2 h, mouse blood was collected and diluted 4 times with EDTA-containing (20 mM) PBS. Diluted whole blood collected from mice with LPS stimulation was stained with CCR2-PE and CD9-APC antibodies for 30 min. Whole blood was further diluted with 5-fold volume of PBS for imaging flow cytometry analysis. An ImageStream MKII flow cytometer (Luminex) was used for imaging the streamed cells/platelets/migrasomes, and IDEAS software (Luminex) was used to measure the number of monocyte migrasomes (CCR2-positive) and platelets (CD9-positive) in the blood from WT and T9 KO mice. Firstly, well-focused events were gated according to the particle Gradient RMS of the bright-field image: the higher the Gradient RMS value, the clearer the focus. Secondly, small particles were gated according to the particle Area of the bright-field image. Thirdly, migrasomes and platelets were gated according to the intensity of CCR2-PE and mouse CD9-APC and analyzed for the number of migrasomes and/or platelets.

### Total membrane proteins isolation

Total membrane proteins, both from plasma membranes and migrasomes, were isolated using the Pierce Cell Surface Protein Isolation Kit (#89881, Thermo Fisher Scientific) following the manufacturer’s instructions. Briefly, cells were cultured in FN-precoated confocal dishes for 12 h. After washing with PBS, cells were treated with Sulfo-NHS-SS-Biotin to label cell membrane proteins with biotin. The biotin-labeled membrane proteins were then isolated from cell bodies or migrasomes using NeutrAvidin Agarose. The total membrane proteins were normalized to equal protein content for western blot analysis.

### Secretion analysis

For in vitro secretion analysis, experiments were conducted following a published protocol with minor modifications^23^. Briefly, equal numbers of the indicated cells were seeded into FN-precoated dishes for 16 h, and the media were gently collected into 50-mL tubes. After double centrifugation at 600× *g* for 10 min at 4 °C, the supernatant was further centrifuged at 2000× *g* for 20 min at 4 °C to remove cell bodies and large debris. Soluble proteins in the medium were concentrated by a 10 KD Amicon filter (Millipore). Meanwhile, migrasomes were purified from the identical cell culture dish, and the cell lysates were collected respectively. Total secretion mixtures of soluble proteins and migrasomes were normalized with the numbers of cells and were then subjected to western blot analysis.

For in vivo secretion analysis, equal volumes of the blood mixture were centrifuged at 600× *g*, 4 °C for 10 min followed by 2000× *g*, 4 °C for 20 min to remove the blood cells. Mouse plasma was centrifuged at 18,000× *g* for 30 min at 4 °C to pellet migrasomes. The supernatant was then concentrated by a 10 KD Amicon filter (Millipore) to collect soluble proteins. Migrasomes (M), soluble proteins (S), and total secretion mixtures (TS) were normalized with the volumes of mouse blood and subjected to western blot analysis.

### Western blot analysis

Proteins from cells or migrasomes were lysed in 2.5% SDS lysis buffer and boiled for 10–20 min at 95 °C. The protein concentration of each sample was determined using the BCA kit. Proteins were resolved on SDS-PAGE gels of appropriate percentages based on the molecular weight of the target proteins, followed by electrophoretic transfer onto PVDF membranes. After blocking with 5% non-fat milk in TBST buffer, membranes were incubated with primary antibodies overnight at 4 °C. Subsequent incubation with secondary antibodies (HRP) for 1 h at room temperature was followed by signal detection using the WESTAR ηC 2.0 kit (CYANAGEN).

The following primary antibodies were used for western blot analyses at the indicated dilution: anti-VAMP2 (1:1000), anti-VAMP3 (1:1000), anti-Rab8a (1:1000), anti-Rab11a (1:2000), anti-SNAP23 (1:2000), anti-TNF-α (1:1000), anti-IL-6 (1:1000), anti-CCR2 (1:1000), anti-CPQ (1:1000), anti-Integrin α5 (1:3000), anti-CD63 (1:1000), anti-CD81 (1:1000), anti-Alix (1:2000), anti-Arf6 (1:1000), anti-Kif23 (1:1000), anti-CD9 (1:1000), anti-Actin (1:5000) and anti-GAPDH (1:5000).

### Statistical analysis

Data analyses, statistical testing, and visualization were conducted in GraphPad Prism 5 (or 8) software. A two-tailed unpaired Student’s *t*-test was applied for the comparison between the two groups. ANOVA and post-hoc tests were used for the comparison of more than two groups. Statistical analysis methods are indicated in the figure legends, and the exact *P*-values are labeled in the figures. The results are presented as means ± SEM.

## Supplementary information

Supplementary Information

Video S1

Video S2

Video S3

Video S4

Video S5

Video S6

Video S7

Video S8

Video S9

## References

[1] Kishimoto, T., Akira, S. & Taga, T. Interleukin-6 and its receptor: a paradigm for cytokines. *Science* **258**, 593–597 (1992). 1411569 10.1126/science.1411569

[2] Ramel, M. C. & Hill, C. S. Spatial regulation of BMP activity. *FEBS Lett.* **586**, 1929–1941 (2012). 22710177 10.1016/j.febslet.2012.02.035

[3] Wartlick, O., Kicheva, A. & Gonzalez-Gaitan, M. Morphogen gradient formation. *Cold Spring Harb. Perspect. Biol.* **1**, a001255 (2009). 20066104 10.1101/cshperspect.a001255 PMC2773637

[4] Kicheva, A., Bollenbach, T., Wartlick, O., Julicher, F. & Gonzalez-Gaitan, M. Investigating the principles of morphogen gradient formation: from tissues to cells. *Curr. Opin. Genet. Dev.* **22**, 527–532 (2012). 22959150 10.1016/j.gde.2012.08.004

[5] Lander, A. D., Nie, Q. & Wan, F. Y. Spatially distributed morphogen production and morphogen gradient formation. *Math. Biosci. Eng.* **2**, 239–262 (2005). 20369921 10.3934/mbe.2005.2.239

[6] Ma, L. et al. Discovery of the migrasome, an organelle mediating release of cytoplasmic contents during cell migration. *Cell Res.* **25**, 24–38 (2015). 25342562 10.1038/cr.2014.135 PMC4650581

[7] Huang, Y. et al. Migrasome formation is mediated by assembly of micron-scale tetraspanin macrodomains. *Nat. Cell Biol.* **21**, 991–1002 (2019). 31371828 10.1038/s41556-019-0367-5

[8] Jiang, D. et al. Migrasomes provide regional cues for organ morphogenesis during zebrafish gastrulation. *Nat. Cell Biol.* **21**, 966–977 (2019). 31371827 10.1038/s41556-019-0358-6

[9] Zhang, C. et al. Monocytes deposit migrasomes to promote embryonic angiogenesis. *Nat. Cell Biol.* **24**, 1726–1738 (2022). 36443426 10.1038/s41556-022-01026-3

[10] Jiao, H. et al. Localized, highly efficient secretion of signaling proteins by migrasomes. *Cell Res.* **34**, 572–585 (2024). 38918584 10.1038/s41422-024-00992-7 PMC11291916

[11] Barlowe, C. et al. COPII: a membrane coat formed by Sec proteins that drive vesicle budding from the endoplasmic reticulum. *Cell* **77**, 895–907 (1994). 8004676 10.1016/0092-8674(94)90138-4

[12] Arvan, P. & Castle, D. Sorting and storage during secretory granule biogenesis: looking backward and looking forward. *Biochem. J.* **332**, 593–610 (1998). 9620860 10.1042/bj3320593 PMC1219518

[13] Davis, A. F. et al. Kinetics of synaptotagmin responses to Ca^2+^ and assembly with the core SNARE complex onto membranes. *Neuron* **24**, 363–376 (1999). 10571230 10.1016/s0896-6273(00)80850-8

[14] Jiao, H. et al. Mitocytosis, a migrasome-mediated mitochondrial quality-control process. *Cell* **184**, 2896–2910.e13 (2021). 34048705 10.1016/j.cell.2021.04.027

[15] Zhao, X. et al. Identification of markers for migrasome detection. *Cell Discov.* **5**, 27 (2019). 31123599 10.1038/s41421-019-0093-y PMC6527679

[16] Kaiser, W. J. et al. RIP3 mediates the embryonic lethality of caspase-8-deficient mice. *Nature* **471**, 368–372 (2011). 21368762 10.1038/nature09857 PMC3060292

[17] Pober, J. S. & Sessa, W. C. Evolving functions of endothelial cells in inflammation. *Nat. Rev. Immunol.* **7**, 803–815 (2007). 17893694 10.1038/nri2171

[18] Amersfoort, J., Eelen, G. & Carmeliet, P. Immunomodulation by endothelial cells - partnering up with the immune system? *Nat. Rev. Immunol.* **22**, 576–588 (2022). 35288707 10.1038/s41577-022-00694-4 PMC8920067

[19] Wu, J. et al. Iterative tomography with digital adaptive optics permits hour-long intravital observation of 3D subcellular dynamics at millisecond scale. *Cell* **184**, 3318–3332.e17 (2021). 34038702 10.1016/j.cell.2021.04.029

[20] Beguier, F. et al. The 10q26 risk haplotype of age-related macular degeneration aggravates subretinal inflammation by impairing monocyte elimination. *Immunity* **53**, 429–441.e8 (2020). 32814029 10.1016/j.immuni.2020.07.021

[21] Zhu, M. et al. Lateral transfer of mRNA and protein by migrasomes modifies the recipient cells. *Cell Res.* **31**, 237–240 (2021). 32994478 10.1038/s41422-020-00415-3 PMC8026638

[22] Jeppesen, D. K. et al. Reassessment of exosome composition. *Cell* **177**, 428–445.e18 (2019). 30951670 10.1016/j.cell.2019.02.029 PMC6664447

[23] Li, S. et al. A new type of ERGIC-ERES membrane contact mediated by TMED9 and SEC12 is required for autophagosome biogenesis. *Cell Res.* **32**, 119–138 (2022). 34561617 10.1038/s41422-021-00563-0 PMC8461442

