# Supplementary material for: Packaged release and targeted delivery of cytokines by migrasomes in circulation
Source: Cell Discov. 2024 Dec 9;10:121. doi: 10.1038/s41421-024-00749-x (PMC11625823; doi:10.1038/s41421-024-00749-x)
Supplement: Supplementary file 1 — Supplementary Information [file 41421_2024_749_MOESM1_ESM.pdf]

Supplementary Information

Supplementary video titles and legends

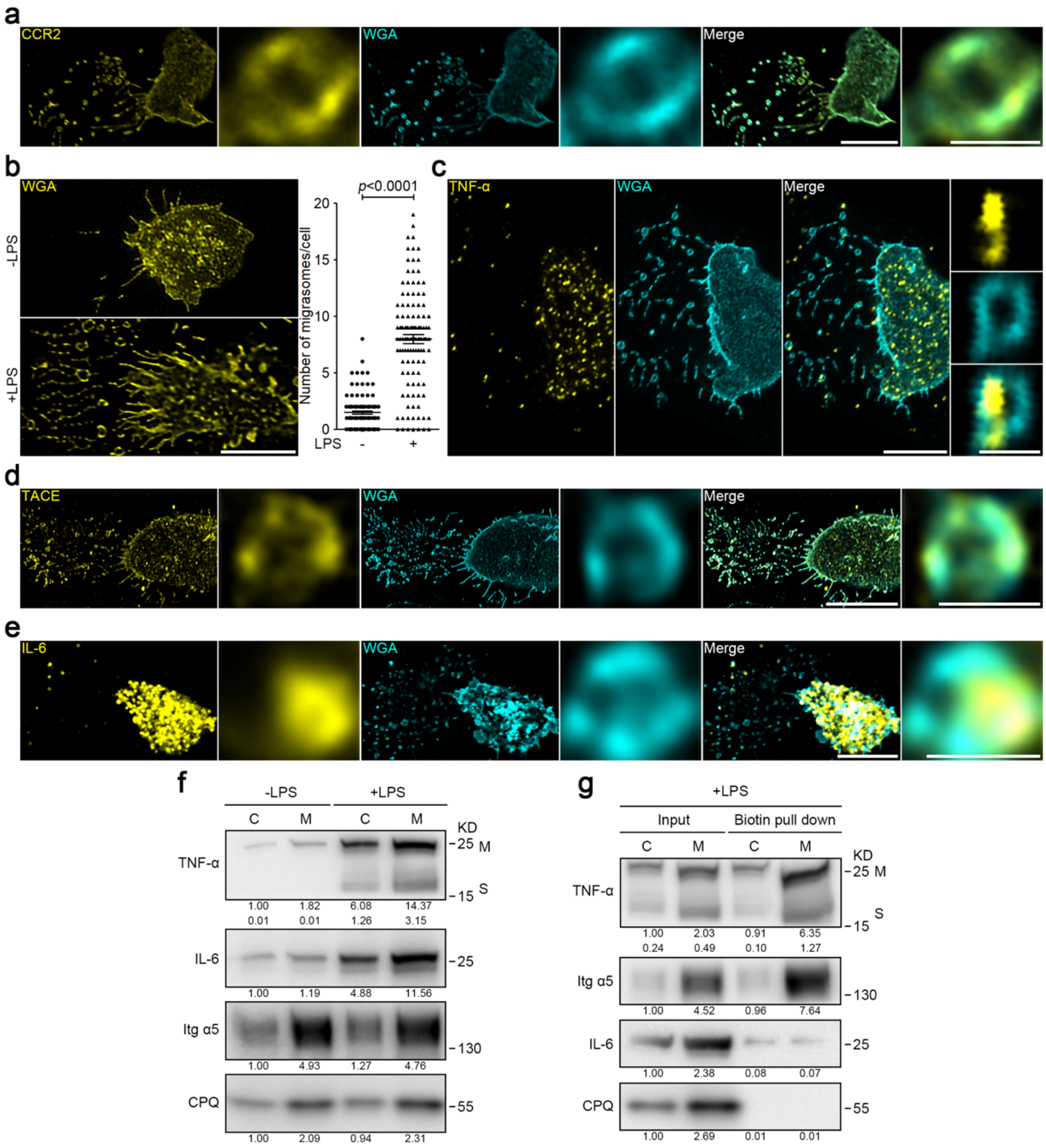

Fig. S1 Cytokines are enriched in monocyte-derived migrasome.

a Mouse monocytes were cultured in fibronectin (FN)-precoated confocal dishes in the presence of 500 ng/mL LPS for 12 hr. Cells were then stained with an anti-CCR2 antibody and WGA before visualization. Scale bar, 5 μm. The right panels show enlarged migrasome. Scale bar, 500 nm.

b Monocytes, treated with or without 500 ng/mL LPS, were stained with WGA before visualization. Scale bar, 5 μm. The right panel shows statistical analysis of the number of migrasome per cell. Error bars, mean ± SEM n > 100 cells from three independent experiments. Two-tailed unpaired t-test was used for statistical analyses.

c-e Immunostaining of endogenous TNF-α (**c**), TACE (**d**) and IL-6 (**e**) in LPS-activated monocytes. Scale bar, 5 μm. Enlarged migrasomes are shown. Scale bar, 500 nm.

f Western blot analysis of migrasomes purified from non-activated and activated monocytes. Equal amounts of total protein from cell bodies (C) and migrasomes (M) were subjected to western blot analysis. Itg α5 and CPQ are used as migrasome markers in monocytes. Both membrane-bound (M) and soluble (S) forms of TNF-α were detected by western blot. Representative densitometry analysis of western blot gray values was shown. Three independent experiments were conducted.

g Western blot analysis of total membrane proteins isolated from monocyte plasma membranes of cell bodies (C) or from migrasomes (M). LPS-activated monocytes were treated with Sulfo-NHS-SS-Biotin to biotinylated membrane proteins. Biotin-labeled membrane proteins were subsequently isolated from cell bodies or migrasomes using NeutrAvidin Agarose, respectively. Cell bodies (C) and migrasomes (M) were normalized to equal total protein loading for western blot analysis using the indicated antibodies. Itg α5 and CPQ are used as migrasome markers in monocytes. Both membrane-bound (M) and soluble (S) forms of TNF-α were detected by western blot. Representative densitometry analysis of western blot gray values was shown. Three independent experiments were conducted.

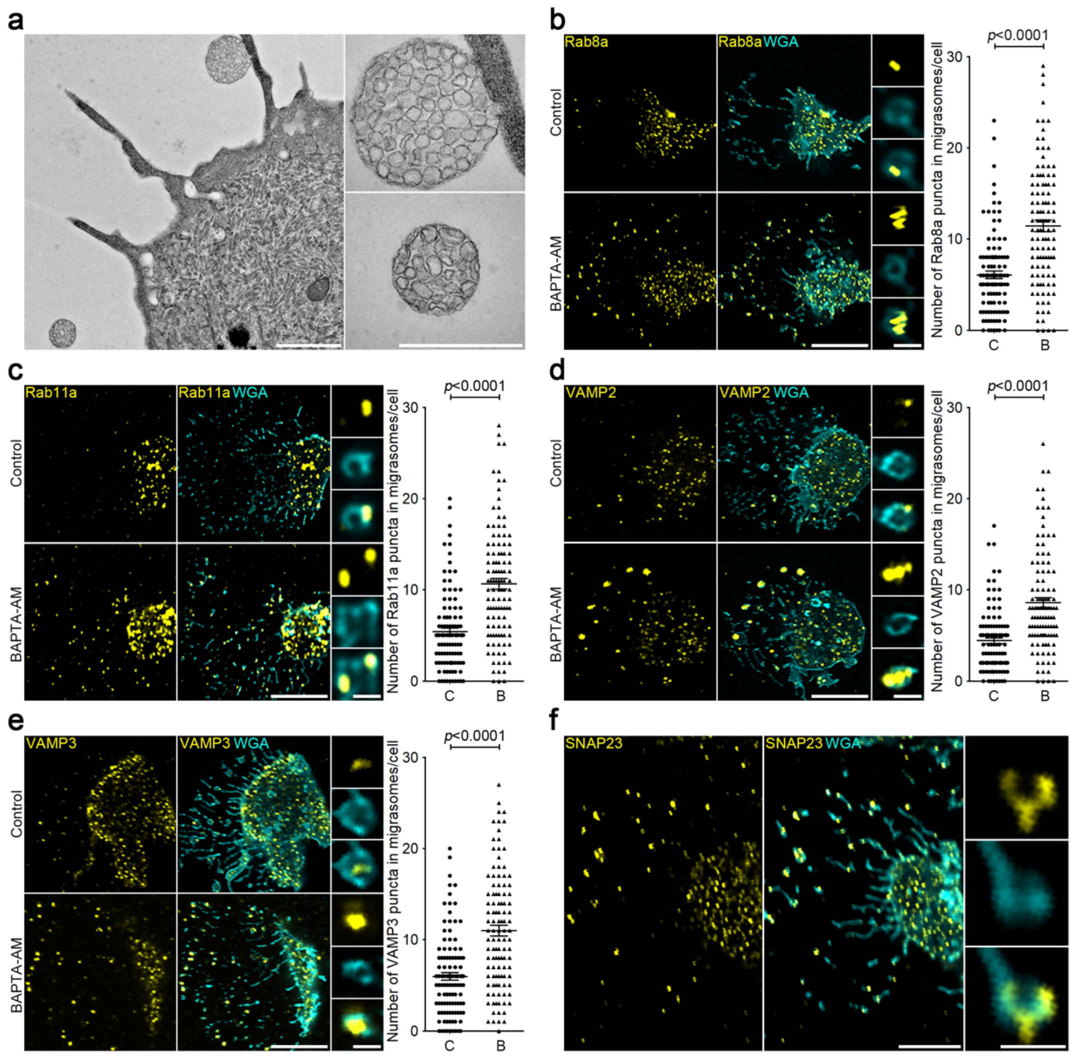

Fig. S2 Secretory carriers are highly enriched in migrasome.

a Representative TEM images of an activated monocyte. Scale bar, 1 μm. Right panels, enlarged migrasomes containing intraluminal vesicles. Scale bar, 200 nm.

b-e LPS-activated monocytes, treated with or without 10 μM BAPTA-AM, were immunostained respectively with antibodies against Rab8a (**b**), Rab11a (**c**), VAMP2 (**d**) and VAMP3 (**e**) before visualization. Scale bar, 5 μm. The right panels show enlarged migrasomes. Scale bar, 500 nm. Statistical analysis of the number of Rab8a (**b**), Rab11a (**c**), VAMP2 (**d**) and VAMP3 (**e**) puncta in migrasomes per cell is shown as the mean ± SEM. n > 100 cells from three independent experiments were analyzed using the two-

tailed unpaired t-test.

f Immunostaining of endogenous SNAP23 in LPS-activated monocytes. Scale bar, 5 μm. Enlarged migrasomes are shown. Scale bar, 500 nm.

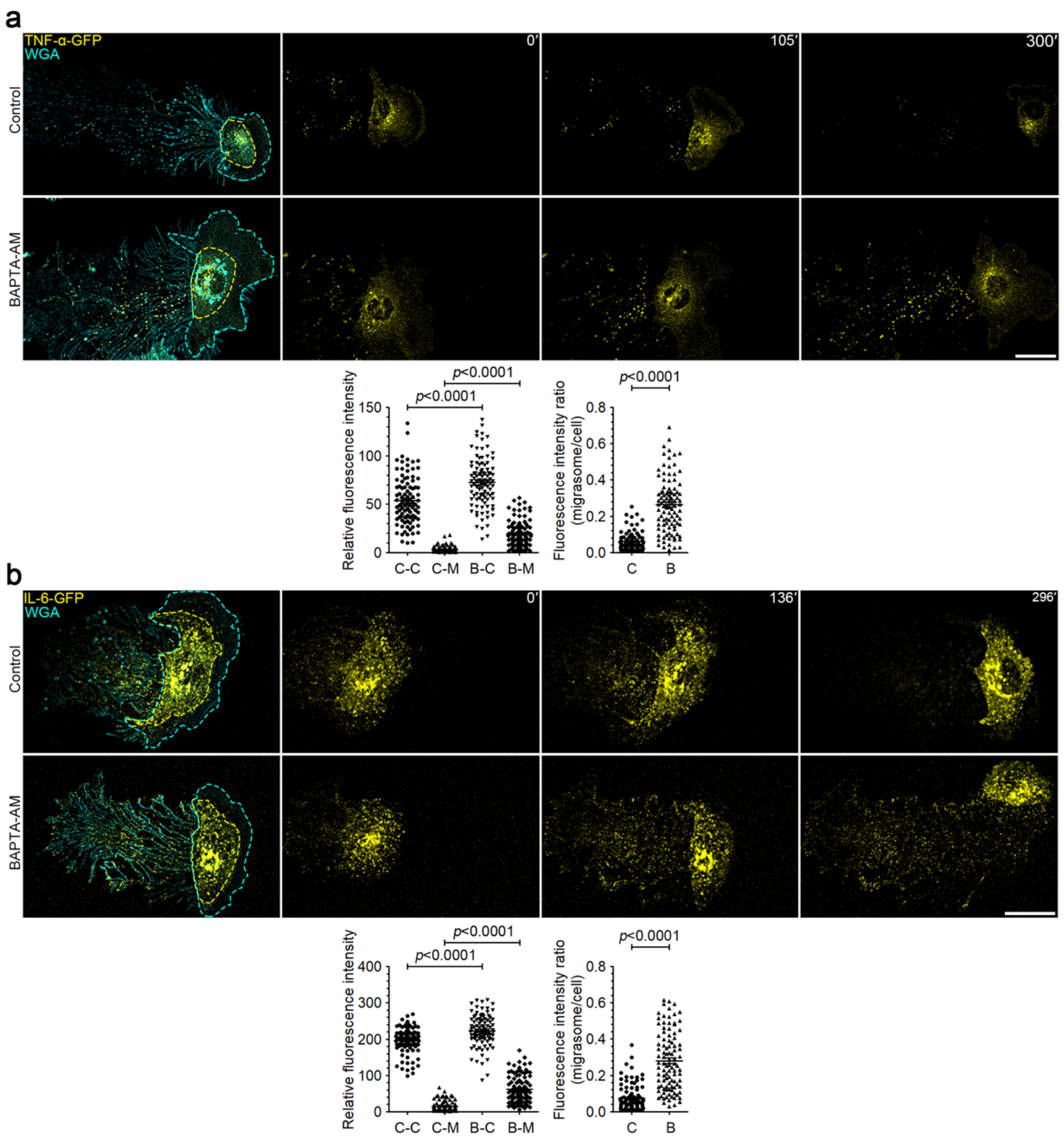

Fig. S3 Migrasomes are the main sites for exocytosis in L929 cells.

a, b L929 cells stably expressing TNF-α-GFP (**a**) or IL-6-GFP (**b**), treated with or without 10 μM BAPTA-AM, were subjected to time-lapse imaging. Time-lapse images were acquired at intervals of 15 min (**a**) or 8min (**b**). Scale bar, 20 μm. Cyan dashed lines outline the cell body. Yellow dashed lines outline TNF-α-GFP vesicles (**a**) or IL-6-GFP vesicles (**b**), respectively. The lower panels show statistical analysis of relative fluorescence intensity of control-cell body (C-C), control-migrasome (C-M), BAPTA-AM-cell body (B-C) and BAPTA-AM-migrasome (B-M). Error bars,

mean ± SEM. n > 50 cells from three independent experiments. ANOVA and Post-hoc tests were used for statistical analyses. Fluorescence intensity ratio of migrasome versus cell body was quantified. Control (C), BAPTA-AM (B). Error bars, mean ± SEM. Two-tailed unpaired t-test was used for statistical analyses.

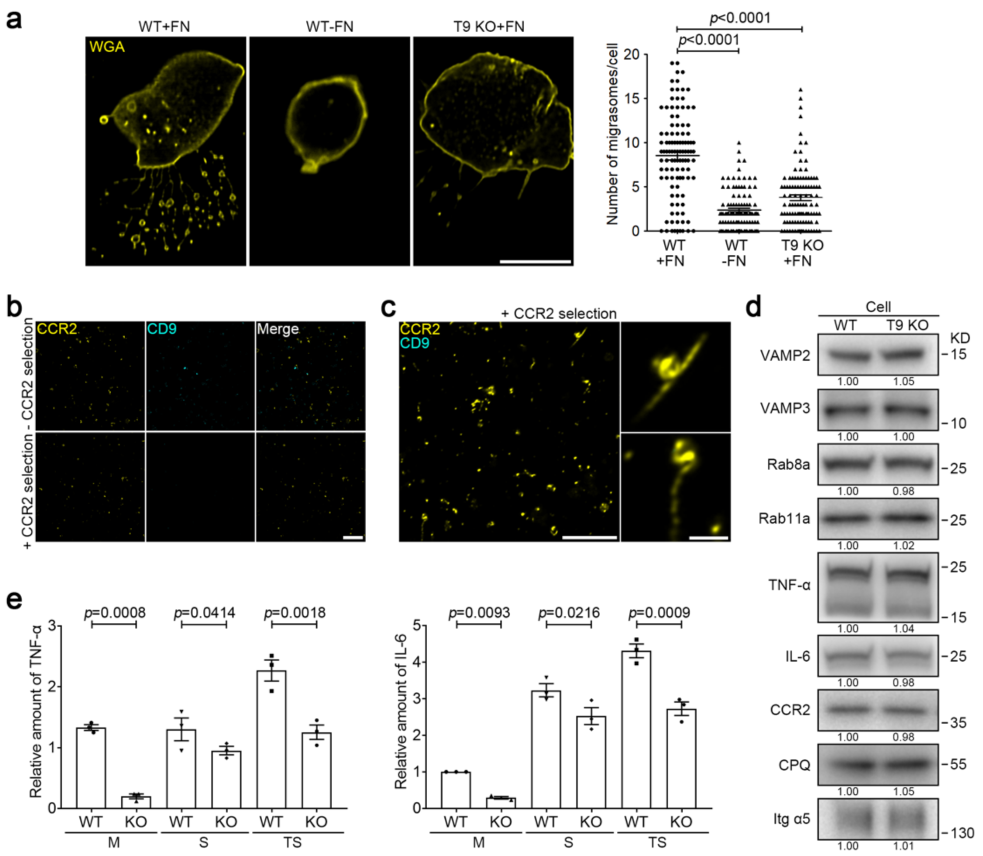

Fig. S4 Tspan 9 regulates monocyte-derived migrasomes and total cytokine levels in mouse blood.

a Confocal images of WT and *Tspan9*$^{-/-}$ (T9 KO) monocytes plated on the indicated dishes in the presence of 500 ng/mL LPS for 12 hr. Scale bar, 5 μm. Quantification of the number of migrasomes per cell is shown as the mean ± SEM. n > 100 cells from three independent experiments were analyzed using the ANOVA and Post-hoc tests (right panel).

b, c Crude migrasomes isolated from blood monocytes were incubated in the CCR2-coated glass, following by washing and immunostaining using the indicated antibodies. Images were acquired by confocal microscopy. Scale bar, 10 μm. The right panels show enlarged migrasomes (**c**). Scale bar, 1 μm.

d Monocytes were isolated from equal volumes of the indicated blood, and then analyzed by western blot using the indicated antibodies. Representative densitometry analysis of western blot gray values was shown. Three independent experiments were conducted.

e Relative amount of TNF-α and IL-6 was quantified corresponding to Fig. **3h** respectively. Quantification is shown as the mean ± SEM from three independent experiments. ANOVA and Post-hoc tests were used for statistical analyses.

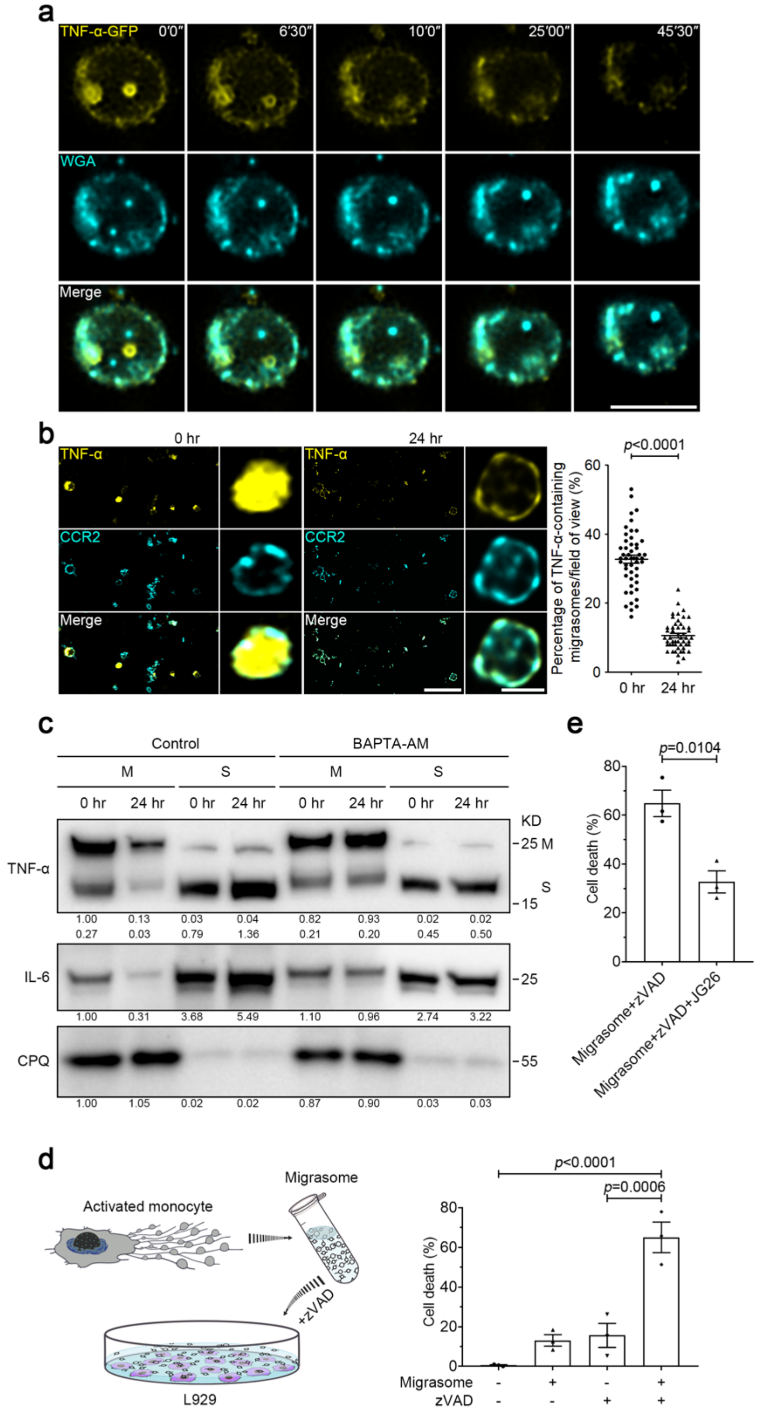

Fig. S5 Sustained release of TNF-α from detached migrasomes.

a Migrasomes purified from L929-TNF-α-GFP cells were incubated in PBS containing 3 mM calcium chloride. After WGA staining, migrasomes were then subjected to time-lapse imaging. Time interval, 30 s. Scale bar, 2 μm.

b Monocyte-derived migrasomes, purified from equal volumes of serum with or without incubation at 20 °C for 24 hr, were immunostained with TNF-α and CCR2 antibodies. Z-stack images were acquired by confocal microscopy, and z-projection was shown as the max intensity. Scale bar, 5 μm. The right panels show enlarged migrasomes. Scale bar, 1 μm. TNF-α-containing migrasomes were quantified and shown as the mean ± SEM from three independent experiments. Two-tailed unpaired t-test was used for statistical analyses.

c Serum collected from mice with LPS treatment was incubated at 20 °C, treated with or without 10 μM BAPTA-AM for 24 hr. Monocyte-derived migrasomes (M) and soluble proteins (S) were purified from equal volumes of the indicated serum sample, and were then subjected to western blot analysis as shown in Fig. **3h**. CPQ is used as migrasome marker in circulating monocytes. Both membrane-bound (M) and soluble (S) forms of TNF-α were detected by western blot. Representative densitometry analysis of western blot gray values was shown. Three independent experiments were conducted.

d L929 cells were cultured in medium containing migrasomes isolated from activated monocytes in the presence of 10 μM zVAD for 18 hr. Cell death was detected by propidium iodide (PI) staining coupled with FACS analysis. The right panel shows statistical analysis of cell death. Error bar, mean ± SEM. Experiments were independently repeated three times. ANOVA and Post-hoc tests were used for statistical analyses.

e PI staining of the indicated L929 cells coupled with FACS analysis as shown in **d**.

Statistical analysis of cell death is shown as the mean ± SEM. Experiments were independently repeated three times. Two-tailed unpaired t-test was used for statistical analyses.

Supplementary video titles and legends

Video S1 Circulating monocyte generates a large amount of extracellular particles in blood.

Intravital imaging of mouse circulating monocytes and monocyte derived extracellular particles. LPS (12 mg/kg) was injected into mice by intraperitoneal (i.p.) injection. After 2 hr, CCR2-PE antibody and WGA-AF647 were injected into mice by intravenous injection (i.v.). Intravital imaging of mouse liver was performed to monitor blood monocytes and monocyte derived extracellular particles. Time-lapse images were acquired at intervals of 90 s. Scale bar, 10 μm. Monocytes and monocyte derived extracellular particles are detected with CCR2-PE antibody, and WGA-AF647 labels blood vessels.

Video S2 TNF-α-laden extracellular particles are shed by circulating monocytes *in vivo*.

Intravital imaging of monocytes in mouse liver after LPS stimulation as shown in Video **S1**. Time interval, 120 s. Scale bar, 10 μm. Monocyte and monocyte derived extracellular particles are labeled with CCR2-PE antibody; membrane-bound TNF-α is detected with TNF-α-AF647 antibody; blood vessels are labeled with WGA-AF488.

Video S3 TNF-α displays a polarized distribution during cell migration.

THP-1 cells were activated in the presence of 100 ng/mL PMA for 24 hr, and were then plated in control or FN-precoated dishes. After TNF-α fluorescent antibody and WGA staining, time-lapse imaging was conducted. Time interval, 180 s. Scale bar, 10 μm.

Video S4 Cell migration causes the polarization of TNF-α vesicles to the rear end of the cell.

L929-TNF-α-BFP cells, treated with or without 10 μM GLPG0187, were subjected to time-lapse imaging. Time interval, 15 min. Scale bar, 20 μm.

Video S5 Cell migration causes the polarization of IL-6 vesicles to the rear end of the cell.

L929-IL-6-GFP cells, treated with or without 10 μM GLPG0187, were subjected to time-lapse imaging. Time interval, 7 min. Scale bar, 20 μm.

Video S6 BAPTA treatment significantly enhanced the level of TNF-α in the migrasomes.

L929-TNF-α-GFP cells, treated with or without 10 μM BAPTA-AM, were subjected to time-lapse imaging. Time interval, 15 min. Scale bar, 20 μm.

Video S7 BAPTA treatment significantly increased IL-6 level in the migrasomes.

L929-IL-6-GFP cells, treated with or without 10 μM BAPTA-AM, were subjected to time-lapse imaging. Time interval, 8 min. Scale bar, 20 μm.

Video S8 Monocytes from *Tspan9-/-* mice produced fewer migrasomes.

Equal numbers of WT and T9 KO monocytes were labeled with anti-CCR2 antibodies conjugated to different colored tags. The color-coded cells were combined for injection into WT mice, and intravital imaging of mouse liver was performed. Time interval, 18 s. Scale bar, 20 μm.

Video S9 Migrasome-bound TNF-α could be released after the migrasomes detach from cells.

Migrasomes purified from L929-TNF-α-GFP cells were incubated in PBS containing 3 mM calcium chloride. After WGA staining, migrasomes were then subjected to time-lapse imaging. Time interval, 30 s. Scale bar, 2 μm.
